# *Trypanosoma cruzi* and Its Soluble Antigens Induce NET Release by Stimulating Toll-Like Receptors

**DOI:** 10.1371/journal.pone.0139569

**Published:** 2015-10-02

**Authors:** Daniel Sousa-Rocha, Mariana Thomaz-Tobias, Larissa Figueiredo Alves Diniz, Priscila Silva Sampaio Souza, Phileno Pinge-Filho, Karina Alves Toledo

**Affiliations:** 1 Department of Biological Sciences, Univ. Estadual Paulista–UNESP (FCL-Assis), Assis, São Paulo, Brazil; 2 Departamento de Ciências Patológicas, Centro de Ciências Biológicas, Universidade Estadual de Londrina, Londrina, Paraná, Brazil; The Hospital for Sick Children and The University of Toronto, CANADA

## Abstract

Neutrophils release fibrous traps of DNA, histones, and granule proteins known as neutrophil extracellular traps (NETs), which contribute to microbicidal killing and have been implicated in autoimmunity. The role of NET formation in the host response to nonbacterial pathogens is not well-understood. In this study, we investigated the release of NETs by human neutrophils upon their interaction with *Trypanosoma cruzi* (Y strain) parasites. Our results showed that human neutrophils stimulated by *T*. *cruzi* generate NETs composed of DNA, histones, and elastase. The release occurred in a dose-, time-, and reactive oxygen species-dependent manner to decrease trypomastigote and increase amastigote numbers of the parasites without affecting their viability. NET release was decreased upon blocking with antibodies against Toll-like receptors 2 and 4. In addition, living parasites were not mandatory in the release of NETs induced by *T*. *cruzi*, as the same results were obtained when molecules from its soluble extract were tested. Our results increase the understanding of the stimulation of NETs by parasites, particularly *T*. *cruzi*. We suggest that contact of *T*. *cruzi* with NETs during Chagas’s disease can limit infection by affecting the infectivity/pathogenicity of the parasite.

## Introduction

Neutrophils are the most abundant leukocytes in the blood and the first to arrive to infection sites, where function in the host defense through phagocytosis and the release of several inflammatory mediators. A landmark study by Brinkmann et al. [1] described a new defense mechanism named neutrophil extracellular traps (NETs), which involves the release of DNA into the extracellular environment associated with various granular and nuclear proteins. NETs can capture and kill many pathogens, including bacteria, fungi, viruses, and parasites [2] such as *Leishmania* spp. and *Toxoplasma gondii* [3, 4]. However, some microorganisms can evade NETs, such as *Vibrio cholera* [5].

Chagas disease, which is caused by *Trypanosoma cruzi* infection, is an important but neglected tropical disease and has emerged as a global public health problem because many *T*. *cruzi*-infected people from Latin America immigrate to countries where the disease is not endemic [6]. An estimated 14,000 people die annually from this disease worldwide [7]. Clinically, *T*. *cruzi* infection causes acute myocarditis followed by chronic cardiomyopathy and vasculopathy in both humans and experimental models. The initial infection control against *T*. *cruzi* is provided by innate immune cells such as macrophages, eosinophils, monocytes, and neutrophils [8]. Interactions between *T*. *cruzi* and phagocytes involve pattern recognition receptors and Toll-like receptors (TLRs) [9, 10].

A large number of studies have demonstrated the effects of NETs and their formation during the capture of bacteria and fungi. However, the role of NETs in the innate immune response against parasites is not well-understood [2]. Although it is known that neutrophils interact with *T*. *cruzi* during the host innate immune response, their role during *T*. *cruzi* infection remains unclear. In addition, the potential of *T*. *cruzi* to induce NETs release is unknown. In this study, we conducted *in vitro* assays and found that *T*. *cruzi* can induce NET release in a dose- and time-dependent manner. Released NETs contain DNA and different proteins, such as histones and elastase. The presence of NETs did not kill the parasite but altered the number of infected cells and the number of released trypomastigote forms. Blocking of TLR–2 and TLR–4 decreased NET release stimulated by both *T*. *cruzi* and its soluble antigens. During *in vivo T*. *cruzi* infection, this mechanism may contribute to the elimination or reduction of the parasitic load.

## Material and Methods

### Ethics statement

All animal procedures were performed in accordance with the guidelines of the Brazilian Code for the Use of Laboratory Animals. The protocols were approved by the Internal Scientific Commission and the Ethics in Animal Experimentation Committee of Londrina State University (Approval Number: CEEA–262/2012). The experimental procedures using human blood were approved by the local Research Ethics Committee of the Faculty of Science and Letters of Assis (Approval Number: CEP–02073912.0.0000.5401). We obtained written informed consent, suggested and approved by the Committee, from each participant before initiating any research procedures.

### Cells

An epithelial cell line (LLC-MK2 original; BCRJ 0146) from *Macaca mulata* was purchased from the Rio de Janeiro Cell Bank (Rio de Janeiro, Brazil). Cells were cultivated in RPMI–1640 medium (Gibco, Grand Island, NY, USA) supplemented with 10% fetal bovine serum (FBS) (Gibco), 2 mM L-glutamine, 0.075% sodium bicarbonate, 100 U/mL penicillin, and 10 mg/mL streptomycin (Invitrogen, Carlsbad, CA, USA) at 37°C in 5% CO_2_. The fetal bovine serum used in this study was inactivated during for 30 min at 70°C [11].

### *Trypanosoma cruzi* parasites

All experiments were performed using the Y strain of *T*. *cruzi* maintained by weekly intraperitoneal inoculation of Swiss mice with 2 × 10^5^ blood trypomastigote forms. Trypomastigote forms were obtained from the supernatants of a previously infected LLC-MK2 cell monolayer by centrifugation at 3000 ×*g* for 30 min at 4^°^C. Heat-killed *T*. *cruzi* were prepared by washing trypomastigotes 3 times in phosphate-buffered saline (PBS), pH 7.2, without calcium and magnesium (Gibco). The cells were resuspended in PBS and incubated in an 80°C water bath for 10 min [12]. Complete loss of viability was verified by counting motile trypomastigotes by light microscopy.

### Soluble antigens from *T*. *cruzi*

Soluble antigens from trypomastigote forms of *T*. *cruzi* were obtained by repeated freezing and thawing [13]. Parasites were washed 3 times with PBS, centrifuged at 800 ×*g* for 10 min, and subjected to 5 freeze-thaw cycles for 1 min at 100°C for 1 min in liquid nitrogen. The remaining material was filtered under aseptic conditions through a 0.22-μm filter; an aliquot was taken to determine protein concentration (BCA Protein Assay Kit; Pierce Biotechnology, Rockford, IL, USA) and stored at −70°C prior to use.

### Isolation of human neutrophils

Whole blood from healthy human donors was collected using plastic sodium Na^+^ heparin Vacutainer blood collection tubes (BD Biosciences, Franklin Lakes, NJ, USA). Neutrophils were isolated the method [14] of Lusisano and Mantovani [15] with some modifications. Cell pellets suspended in Hank´s balanced salt solution (HANKS) containing 0.1% gelatin (w/v) (HANKS-gel) were > 90% viable as determined by the Trypan blue exclusion test, and 90–95% of cells were found to be neutrophils.

### Immunofluorescence microscopy

Neutrophils were incubated on poly-l-lysine-treated glass coverslips in a 24-well plate with HANKS, *T*. *cruzi*, or its soluble antigen followed by incubation at 37°C for 4 h. Samples were fixed with 3% paraformaldehyde for 20 min at room temperature and then blocked in PBS containing 10% skim milk for 1 h at room temperature. Coverslips were incubated overnight with anti-elastase (SAB2100672) and anti-histone (SAB4500354) primary antibodies (Sigma-Aldrich, St. Louis, MO, USA) followed by fluorescein isothiocyanate-conjugated anti-IgG mouse secondary antibody (AP187F) (Millipore Corporation, Billerica, MA, USA) for 30 min at 37°C. After rinsing in PBS, coverslips were washed and mounted with ProLong Antifade containing DAPI (Invitrogen). Images were collected using a Nikon Eclipse Ti fluorescence microscope equipped with the Nikon Sight digital camera with NIS-Elements software (Nikon Corporation, Tokyo, Japan).

### DNA measurement

Neutrophils (2 × 10^5^) were incubated with HANKS, phorbol myristate acetate (PMA), and parasites or their soluble antigens. At various time points, the cells were centrifuged at 200 ×*g* for 8 min before adding 500 mU/mL micrococcal nuclease (Worthington Biochemical, Lakewood, NJ, USA). The cultures were incubated for 10 min at 37°C to obtain the NET solution. Enzymatic digestion was terminated by using 5 mM EDTA, and cultures were centrifuged at 200 ×*g* for 8 min. Twenty microliters of cell-free supernatant were used for quantification of double-stranded DNA using the dsDNA High Sensibility Assay (Invitrogen) following the manufacturer recommendations. Extracellular DNA was measured using a Qubit 2.0 (Invitrogen) fluorometer. In the same assays, 20 μM diphenyleneiodonium chloride (DPI, Sigma-Aldrich) was added and the samples were incubated for 30 min at 37°C to inhibit NADPH oxidase. The DNA profile was evaluated in a 1.5% agarose gel prepared in Tris-acetate-EDTA buffer and containing GelRed (1:10,000) (Biotium, Hayward, CA, USA). Electrophoresis was conducted at 120 V for 2 h and DNA was visualized using an ultraviolet transilluminator. The 1-kb DNA ladder was purchased from Kasvi (Curitiba, PR, Brazil).

### Elastase activity

Elastase enzyme activity in NET samples was measured using an enzymatic colorimetric method with the substrate *N*-methoxysuccinyl-Ala-Ala-Pro-Val *p*-nitroanilide (Sigma-Aldrich). NET solution and 1 mM elastase substrate were mixed. After 30 min, absorbance was measured at 405nm in a microplate reader. Various concentrations of purified elastase enzyme from human neutrophils (EMD Chemicals Inc., Billerica, MA, USA) were used as standards.

### Detection of citrullinated H3 by immunoblotting

Total NET solution was quantified using a bicinchoninic acid protein assay kit (Pierce). Next, 30 μg total protein was diluted in sample buffer (62.5 M Tris, pH 6.8, 2% SDS (w/v), 5% glycerol (v/v), 30 μM phenol red, and 0.9% β-mercaptoethanol) and incubated for 5 min at 100°C. Samples were resolved in 15% polyacrylamide gels and transferred onto nitrocellulose membranes (Amersham Biosciences, Amersham, UK). After blocking in 5% non-fat dry milk, the membranes were probed with primary antibody against citrullinated H3 (clone ab5103, Abcam, Cambridge, UK) for 2 h at room temperature. After washing, the membranes were incubated with horseradish peroxidase-conjugated donkey secondary anti-mouse IgG (Jackson Immunoresearch, West Grove, PA, USA) for 45 min at room temperature. Bound antibodies were revealed by enhanced chemiluminescence using an ECL kit (Pierce). Densitometry analyses were done using ImageJ software.

### Viability of parasites

*Trypanosoma cruzi* (10^4^ parasites) was incubated in HANKS or NET solution containing 300 ng DNA and induced by 25 nM PMA. After 1 h, parasite viability was assessed by counting motile trypomatigotes in a Neubauer chamber [3]. Samples were counted in pure form or were diluted to ensure that all trypomastigotes were counted only once. Counts were conducted in triplicate.

### Infectivity and release of *T*. *cruzi* from LLC-MK2 cells

*Trypanosoma cruzi* (10^4^ parasites) was incubated in HANKS or 300 ng DNA NET solution induced by 25 nM PMA as described above. After 1 h, parasites were added to LLC-MK2 cells in monolayer culture in a 24-well plate and then incubated for 2 h at 37°C. Cultures were maintained for 5 days in RPMI medium supplemented with 5% fetal bovine serum. The number of trypomastigote forms in the supernatant was assessed by counting in a Neubauer chamber. The number of infected cells was counted by microscopic observation using the well diameter as a parameter. After incubation, the viability of LLC-MK2 cells was evaluated using the MTT assay. Cells were incubated for 4 h in the presence of 0.5 mg/mL MTT salt. Later, 100 μL DMSO was added to dissolve formazan crystals and absorbance was measured in a spectrophotometer at 570 nm.

### *In vivo T*. *cruzi* infection

Swiss male mice were injected intraperitoneally with 1000 trypomastigote forms previously treated with 300 ng NET or HANKS for 1 h. Parasitemia was regularly determined in tail blood as previously described [16].

### Analysis of oxidative burst

Neutrophils were seeded at 2.5 × 10^5^ cells/100 μL in a 96-well plate and resuspended in HANKS in the presence of 2′,7′-dichlorofluorescein diacetate (Sigma) in a final concentration of 100 μM. Cells were stained for 20 min and centrifuged for 5 min at 370 ×*g*. The supernatant was removed and the cells were stimulated with 25 nM PMA, *T*. *cruzi* (5 *T*. *cruzi* or Tc: 1 Neutrophil or Ne), 50 μg/mL soluble antigens, or HANKS. Fluorescence was measured after 30 min at 485 nm excitation and 520 nm emission in a plate reader FLx800 (Biotek, Winooski, VT, USA). DPI (20 μM) was incubated with the samples for 30 min at 37°C to inhibit NADPH oxidase.

### Role of TLR2 and TLR4 in NET release induced by *T*. *cruzi*

Neutrophils were preincubated for 1 h at 37°C with the monoclonal antibodies anti-TLR4 and anti-TLR2 (20 μg/mL) (0.5 μg; Pab-hTLR2 and Pab-hTLR4) (Invivogen, Carlsbad, CA, USA) before stimulation with *T*. *cruzi*. Anti-TLR2 and anti-TLR4 were mouse anti-human IgG antibodies, and we used a mouse IgG isotype in all control wells. To washed cells, we added *T*. *cruzi* or its soluble antigens and incubated the cells for 4 h at 37°C. Released NETs were recovered by enzymatic digestion with 500 mU/mL micrococcal nuclease. The free-cell supernatant containing 5 mM EDTA was used for double-stranded DNA measurement using the dsDNA High Sensibility Assay as described above. The effectiveness in blocking TLR2 and TLR4 receptors was assayed by measuring chemokine MIP-β in the supernatant of Pam2CSK4- and lipopolysaccharide (LPS)-stimulated neutrophils. Pam2CSK4 (10 ng/mL) and LPS (10 ng/mL) were purchased from Invivogen. MIP–1β was measured using R&D Systems DuoSet enzyme-linked immunosorbent assays (Minneapolis, MN, USA).

### Statistical analyses

Statistical analyses were performed by using Prism software (GraphPad Software, Inc., La Jolla, CA, USA). One-way analysis of variance (ANOVA) and Bonferroni post-test analysis were used to determine statistical significance. *P* values of < 0.05 were considered significant.

## Results and Discussion

### Trypomastigotes, its soluble antigens, and even heat-killed forms induce NETs formation by human neutrophils

NETs were described as a host defense mechanism of the innate immune response. NETs are composed of DNA associated with nuclear and granular proteins (histones, elastase, myeloperoxidase, pentraxin, lactoferrin, and others); numerous pathogens induce NETs [2, 17]. Here, we demonstrated that *T*. *cruzi* induces NET release from human neutrophils using fluorescence microscopy, DNA and elastase quantification, and infection protocols.

**Fig 1:**
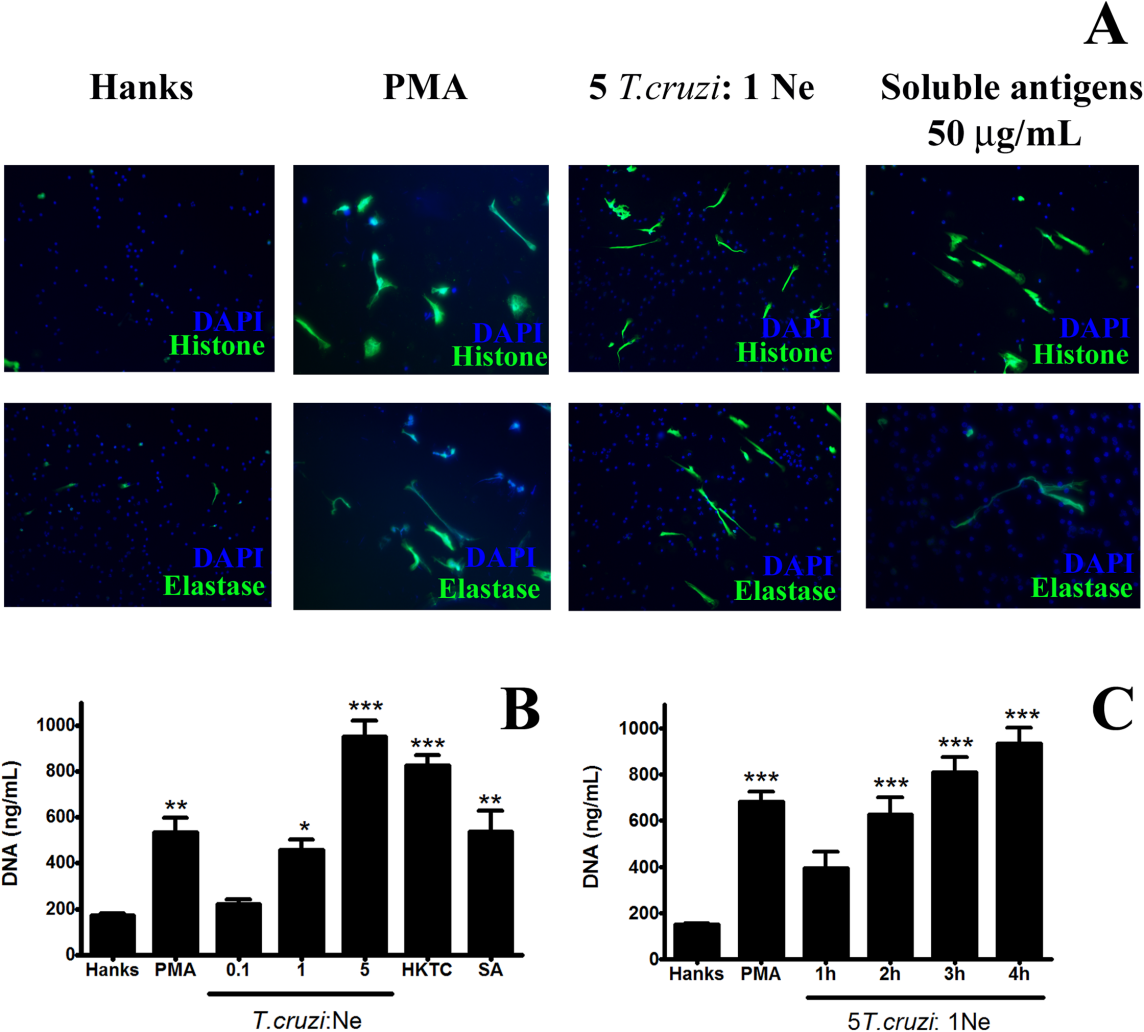
*Trypanosoma cruzi* parasites and its soluble antigens induce NET release. Neutrophils (2 × 10^5^) were incubated with trypomastigote forms or their soluble antigens for 1–4 h and analyzed for NETs release by fluorescence microscopy and extracellular DNA quantification. **(A)** Neutrophils were incubated with *T*. *cruzi* (5 Tc: 1 Ne), soluble antigen (50 μg/mL), PMA (25 nM), or only HANKS for 4 h. NETs were observed by fluorescence staining using antibodies: anti-histone (green), anti-elastase (green), and fluorescein isothiocyanate-conjugated antibody and DAPI (blue). Stimulated neutrophils showed 5 ± 4 NETs by field (40× objective). **(B)** Neutrophils were incubated with *T*. *cruzi* in different ratios (0.1–5 parasites: 1 Ne), heat-killed *T*. *cruzi* (5 parasites: 1 Ne), and soluble antigen (50 μg/mL) for 4 h. HANKS and PMA (25 nM) were used as negative and positive controls, respectively. DNA in the supernatants was quantified using a dsDNA High Sensibility Assay Kit. **(C)** Neutrophils were incubated with *T*. *cruzi* (5 Tc: 1 Ne) for different time periods (1–4 h). HANKS and PMA (25 nM) were used as negative and positive controls, respectively. DNA in the supernatants were quantified as described in B. All experiments were conducted in triplicate with at least 3 independent assays. The results (B, C) were analyzed by ANOVA followed by Bonferroni multiple comparisons test. Asterisks indicates significant differences when compared with the control group (HANKS) (*P < 0.05, **P < 0.01, ***P < 0.001).

Initially, to determine whether *T*. *cruzi* triggers the release of NETs, neutrophils were incubated with trypomastigote forms (5 *T*. *cruzi*: 1 Ne ratio) for 4 h followed by DNA analysis using (DAPI; blue) and histone and elastase (specific antibodies; green) staining [18]. Based on fluorescence microscopy (**Fig 1A**), neutrophils incubated with only HANKS buffer showed multilobulated nuclei without extracellular projections and minimum staining for histone and elastase. In contrast, neutrophils incubated with *T*. *cruzi* had several extracellular DNA projections, which were stained with specific antibodies against histone and elastase. Similar results were obtained when human neutrophils were incubated with soluble antigens from trypomastigote forms of *T*. *cruzi* or PMA (**Fig 1A**). Separated images containing nucleus and protein staining are shown in **S1 Fig**. Weak intracellular staining, even in non-stimulated neutrophils, was also observed in previous studies [18–21]. In contrast, some authors showed that paraformaldehyde fixation is sufficient to allow for antibody detection of intracellular elastase [16, 22, 23]. In previous studies, different antibodies from several manufacturers were used; however, these studies did not include product codes, hindering comparison of the results.

In order to better describe this effect, extracellular DNA was measured in the supernatant from neutrophils incubated with different ratios of *T*. *cruzi* and neutrophils for varying periods of time (**Fig 1B and 1C**). After 4 h of incubation, *T*. *cruzi* induced the release of double-stranded DNA from the 1 *T*. *cruzi*: 1 Ne ratio, with the maximum release observed for the 5 *T*. *cruzi*: 1 Ne ratio. Interestingly, the use of a 5:1 heat-killed *T*. *cruzi*:neutrophil ratio and soluble antigens induced release of DNA similar to that seen for live *T*. *cruzi*. The presence of autologous serum from neutrophil donors did not affect *T*. *cruzi*-induced NET formation (data not shown).

These results indicate that *T*. *cruzi* can stimulate NET release during the invasion of host cells, which may be triggered by soluble factors derived from extracellular living or dead parasites. Similar results were obtained in previous studies for *T*. *gondii* [4] and *Leishmania amazonensis* [3].

**Fig 2:**
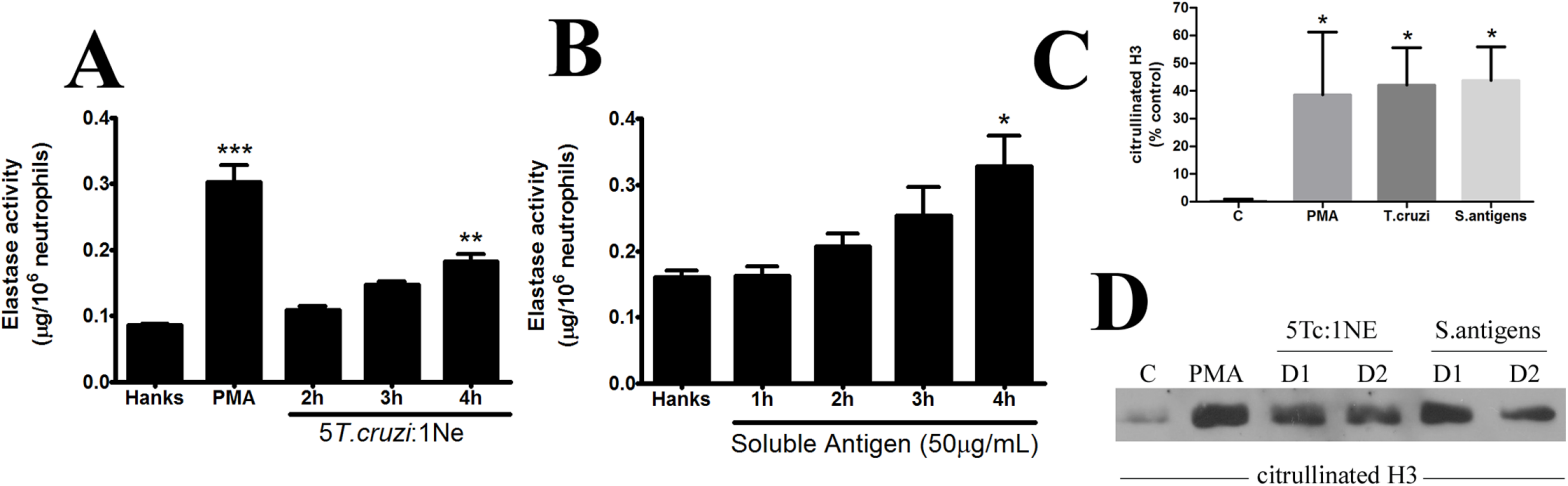
NETs released by *T*. *cruzi* and its soluble antigens containing granular and nuclear proteins. Neutrophils (2 × 10^5^) were incubated in a ratio of 5 *T*. *cruzi*: 1 neutrophil **(A)** or its soluble antigens **(B)** for 1–4 h. The supernatant from these cells containing isolated NETs was analyzed for elastase activity using an enzymatic colorimetric method with the substrate *N*-methoxysuccinyl-Ala-Ala-Pro-Val *p*-nitroaniline (1 mM). HANKS and PMA (25 nM) incubated for 4 h were used as negative and positive controls, respectively. **(C)** Densitometry analyses from three independent immunoblottings for citrullinated H3 (3 donors for control and PMA; 4 donors for *Tcruzi* and soluble antigens). **(D)** NET solution from 2 different donors (D1 and D2) stimulated with *T*. *cruzi* (5 Tc: 1 Ne) or its soluble antigens (50 μg/mL) for 4 h was subjected to identification of citrullinated H3 by immunoblotting. All experiments were conducted in triplicate with at least 3 independent assays. The results (A, B and C) were analyzed by ANOVA followed by Bonferroni multiple comparisons test. Asterisks indicates significant differences when compared with the control group (HANKS) (*P < 0.05, **P < 0.01).

The protein composition of NETs generated by neutrophils incubated with *T*. *cruzi* and its soluble molecules was determined by measuring released elastase (**Fig 2A and 2B**) and specific immunostaining with citrullinated histone–3 (**Fig 2C and 2D**). Neutrophils incubated with *T*. *cruzi* (5 Tc: 1 Ne) for 4 h increased the release of elastase by 1.73-fold into the supernatant compared to in the negative control, HANKS (**Fig 2A**). This amount was lower than the release observed for PMA, the positive control. Soluble antigens from *T*. *cruzi* also induced elastase release by a lower amount (1.48-fold) (**Fig 2B**). The presence of citrullinated H3 [24] from *T*. *cruzi* and soluble antigen stimulated-neutrophils were confirmed in our analysis from different donors (D1 and D2) (**Fig 2C and 2D**). The induction of citrullination of histone by PMA is controversial [18, 25–27]. On our hands, neutrophils incubated with PMA but not HANKS showed immunostaining for citrullinated H3. The presence of citrullinated H3 may be associated with NETose processes [28]. Together, these results (**Figs 1 and 2**) support that neutrophils incubated with *T*. *cruzi* or its soluble antigens receive sufficient stimuli to generate “classic NETs”, with the main structure consisting of DNA which is "decorated" by nuclear and granular proteins such as histone and elastase. In the pathogenesis of Chagas disease, the extracellular presence of granular proteins, mainly myeloperoxidase, can contribute to myoblast injury [27]. Thus, NETs release induced by *T*. *cruzi* from infiltrated neutrophils in the cardiac lesions may increase tissue damage.

### NETs does not kill *T*. *cruzi* but interferes with its infection ability

**Fig 3:**
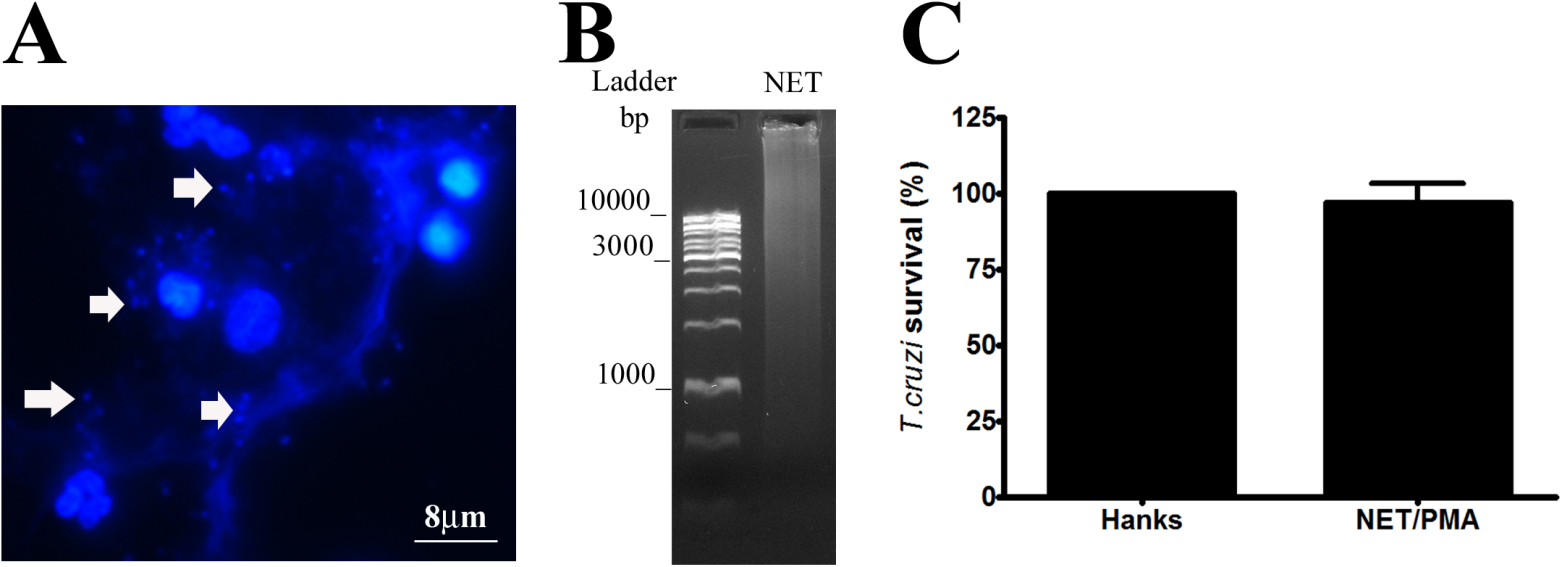
NETs were not able to kill *T*. *cruzi*. **(A)** Neutrophils were incubated with *T*. *cruzi* (10 Tc: 1 Ne) for 4 h. NETs were observed by fluorescence staining using DAPI (blue). White arrows indicate trapped *T*. *cruzi* in the NETs. **(B)** NET solution (1 μg) was evaluated by 1.5% agarose gel electrophoresis. NET solution and ladder DNA (1 kb) were stained using GelRed (1:10,000). **(C)** *Trypanosoma cruzi* (10^4^ parasites) were incubated with NET solution or HANKS for 1 h at 37°C. Motile parasites were counted in a Neubauer chamber as live parasites. The results are shown as *T*. *cruzi* survival (%), where HANKS results were considered to be 100%. The results were analyzed by Student’s *t*-test and did not reveal significant differences. All experiments were conducted in triplicate in independent assays.

NETs are structures that immobilize a broad range of pathogens, but it is unknown whether microorganisms immobilized by NETs are dead [29]. When neutrophils were stimulated by *T*. *cruzi* (10 Tc: 1 Ne), some parasites were captured by the NETs (**Fig 3A**; white arrows). This result was similar that observed by Guimarães-Costa et al. [3]. We examined whether NETs generated from neutrophils stimulated by PMA could kill the parasite *T*. *cruzi*. Thus, parasites were incubated for 1 h in the supernatant containing NETs. The NETs profile was evaluated by agarose electrophoresis as shown in **Fig 3B.** The NETs solution contained large heterogeneous fragments greater than 1,000 base pairs (bp). Parasite viability was assessed by observing their mobility using a light microscope. Under these conditions, NETs could not kill the parasite *T*. *cruzi*, as their mobility was similar to that found in parasites incubated with HANKS solution only (**Fig 3C**).

**Fig 4:**
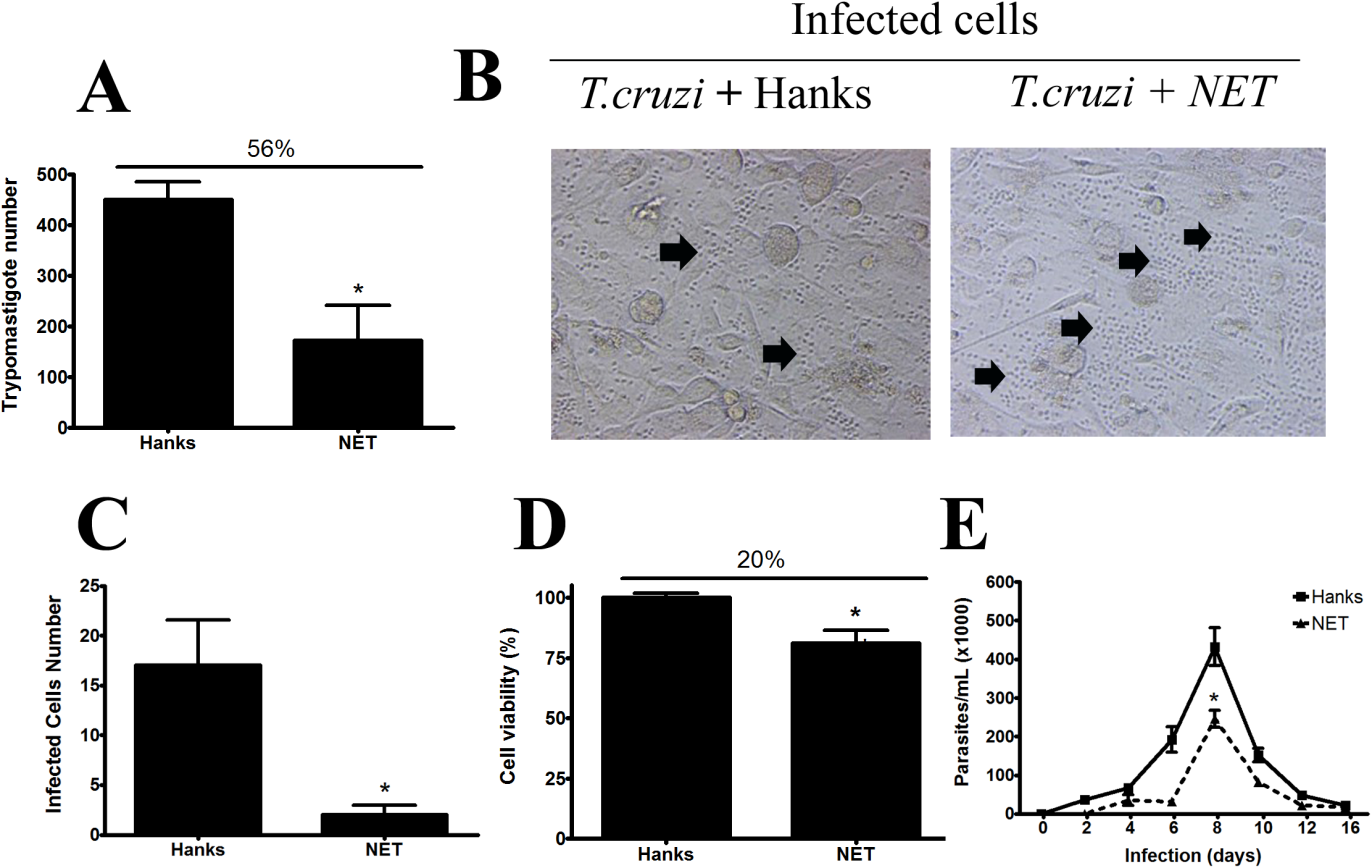
NETs decrease *T*. *cruzi* infectivity. **(A–D)** LLC-MK2 cell (2 × 10^4^) monolayers were infected with trypomastigotes (10^5^) pretreated for 1 h with NET solution released from neutrophils stimulated with 25 nM PMA (NETs) or only HANKS. After 5 days, we evaluated: **(A)** the total number of released trypomastigotes in the supernatant, **(B)** the presence of amastigote forms (black arrows), **(C)** the number of infected cells by light microscopy traversing the diameter of each well, and **(D)** the viability of cells using the MTT assay where cells cultured with HANKS correspond to 100% viability. **(E)** Mice (N = 5) were infected with trypomastigotes (1000 forms) pretreated with NET or HANKS. Parasitemia was monitored in the blood for 16 days. All experiments were conducted 3 times in independent assays. The results show the media ± SD. Asterisks indicate significant differences compared with the control group (HANKS) (*P < 0.05).

We evaluated whether contact with NETs could result in a decrease in parasite pathogenicity, such as whether other microorganisms are killed and/or lose their virulence when captured by NETs [17]. For this purpose, LLC-MK2 cells were infected with *T*. *cruzi* that had been pretreated with NETs or HANKS solution. After 5 days, the number of trypomastigotes (infective form; **Fig 4A**) and the presence of amastigotes (non-infective form; **Fig 4B**) were counted in the supernatant of the cells. In the supernatant of HANKS *T*. *cruzi* infected-cells, we counted a large number of trypomastigote forms. Additionally, we observed a low frequency of amastigote forms, identified as rounded parasites without motility (see the black arrows in **Fig 4B and S1 Movie**). In contrast, when the parasites were pretreated with NETs, their cultures showed low numbers of trypomastigote forms (**Fig 4A and S2 Movie**) and large numbers of amastigote forms (see the black arrows in **Fig 4B and S2 Movie**).

The number of infected cells was calculated based on the traversing vertical axis from each well of a 24-well plate (**Fig 4C**). There was a large number of infected cells when the parasites were pretreated with HANKS, but this number decreased when NET solution was used as a pretreatment. These results can be also observed in the **S1 and S2 Movies (Supporting Information)**. The presence of NETs in the medium decreased cell viability by 20% as shown in **Fig 4D**. However, the loss of viability/infectivity of *T*. *cruzi* was much larger (56%; 2.8-fold in comparison) (**Fig 4A**). This difference suggests that the decrease in viability/infectivity of the parasite reflects other characteristics in addition to cellular viability.

Our results demonstrated that NETs could not kill the *T*. *cruzi* parasite. This differed from the results for *L*. *amazonensis*, in which parasites were co-incubated for 2 h in the presence of NETs [3], and *Toxoplasma gondii* treated with NETs for 4 h [4]. These differences may be because of the different times and stimuli used in each protocol or differences in the studied pathogen. However, our results are similar to studies examining *Leishmania donovani* [30] *Staphylococcus aureus* and *Candida albicans* [29], and *Neisseria meningitidis* [31], which showed that NETs could not kill these microorganisms.

The effect of NETs on *T*. *cruzi* infection was evaluated *in vivo* (**Fig 4E**). Mice were intraperitoneally infected with *T*. *cruzi* pretreated or not with NETs and the presence of parasites in the blood was monitored for 16 days. Parasitemia increased until 8 days and then declined in both groups. Pretreatment of parasites with NETs resulted in a significantly decreased number of parasites in the blood (**Fig 4E**). Boari et al. demonstrated that spleen neutrophils purified from *T*. *cruzi*-infected mice produced high levels of interleukin–10 and low levels of tumor necrosis factor when they were re-stimulated [32]. Under these conditions, mice showed increased parasitemia but decreased mortality, as liver damage was less severe. The authors suggest that in the reduced parasitemia was not sufficient to prevent mortality when important tissues were damaged. They proposed that neutrophils play a regulatory role in exacerbated Th1 inflammatory responses. Based on our results, we hypothesize that during *T*. *cruzi* infection, inflammatory and non-inflammatory infiltrated neutrophils can be found in the cardiac tissue. This balance can prevent excessive host tissue damage but also favors chronicity of the infection.

The events observed when the *T*. *cruzi* was incubated with NETs suggest a loss of virulence accompanied by decreased invasiveness, replication, and/or cell release. Abi et al. demonstrated that contact between *T*. *gondii* and NETs results in parasite death [4]. NETs are composed of DNA and several proteins, such as elastase, cathepsin D, histones, and myeloperoxidade, among others [33]. This gives to NETs the ability to degrade many virulence factors, as shown for IpaB from *Shigella flexneri* and alpha-toxin from *S*. *aureus* [1]. The virulence factors from *T*. *cruzi* may be affected by microbicidal components from NETs. Among them, gp82, gp85, gp63 and others are related to cell invasion [34]. Additionally, NET compounds may modulate the role of other important cells during *T*. *cruzi* infection. Luna-Gomes et al. [20] found that neutrophil elastase appears to be involved in increased trypanocidal activity. The addition of purified elastase reduced trypomastigote release by infected macrophages, in which the production of nitric oxide and tumor necrosis factor-alpha was stimulated.

### NET release induced by *T*. *cruzi* is a reactive oxygen species (ROS)-dependent process

The molecular mechanisms involved in the process of NETose described thus far indicate the importance of NADPH activation [21, 23]. Neutrophils from patients with chronic granulomatous disease showed a decreased ability to release NETs [22]. Phagocytosis of parasites, including *T*. *cruzi*, induces immune cells to release ROS, which are important for the killing of these organisms (reviewed [25]). The NADPH activation requirement was investigated during NET release by *L*. *donovani* [30] and *Bernoitia besnoiti* [26] stimulation. Both parasites induced the NETosis process in an ROS-dependent manner.

**Fig 5:**
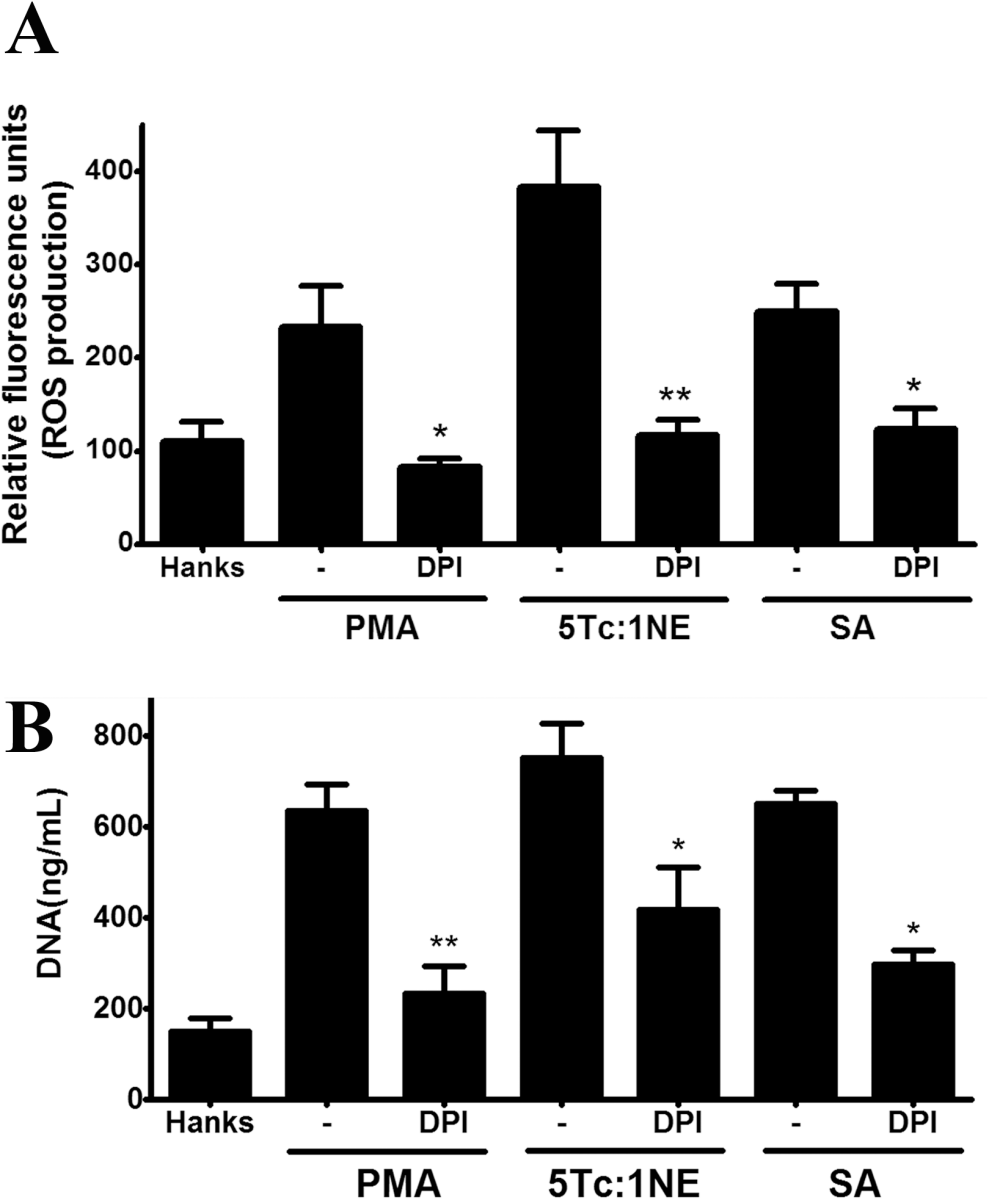
NET release induced by *T*. *cruzi* is an ROS-dependent process. **(A)** 2′,7′-Dichlorofluorescein-labeled neutrophils (2.5 × 10^5^) were pretreated or not with DPI (20 μM) and stimulated by PMA (25 nM), *T*. *cruzi* (5 Tc:1 Ne), soluble antigens (50 μg/mL), or HANKS for 30 min. ROS production was measured by fluorescence. **(B)** Neutrophils (2.5 × 10^5^) pretreated or not with DPI (20 μM) were stimulated by PMA (25 nM), *T*. *cruzi* (5 Tc: 1 Ne), soluble antigens (50 μg/mL), or HANKS for 4 h. Extracellular DNA was measured in the supernatant using the dsDNA High Sensibility Assay Kit. The results were analyzed by ANOVA followed by Bonferroni multiple comparisons test. Asterisks indicate significant differences compared with the control group (HANKS) (*P < 0.05, **P < 0.01).

We evaluated ROS production and NETs release stimulated by *T*. *cruzi* or its soluble antigens in neutrophils pretreated with DPI, an NADPH inhibitor. After 30 min, *T*. *cruzi* induced ROS production, which was inhibited in the presence of DPI (**Fig 5A**). Similar results were obtained when neutrophils were stimulated with PMA or soluble antigens from *T*. *cruzi*. Additionally, the blocking of NADPH decreased NET release induced by *T*. *cruzi*, its soluble antigens, and PMA (**Fig 5B**). These data indicate that the *T*. *cruzi* parasite stimulates NET release in an ROS-dependent process.

### *Trypanosoma cruzi* induces NET release by activating TLRs

Neutrophils detect the presence of a pathogen, particularly through TLRs [35]. TLRs are transmembrane receptors that perform an important role in innate immunity by recognizing pathogens. The recognition of conserved molecular patterns from microorganisms by these receptors leads to a signal transduction cascade that results in cell and cytokine activation. The expression and function of TLRs in neutrophils has been investigated; neutrophils express TLRs 1, 2, 4, 5, 6, 7, 8, 9, and 10 [36]. Many molecules from trypomastigotes, including glycosylphosphatidylinositol anchor [9] and DNA [37], can stimulate the synthesis of pro-inflammatory cytokines. Specifically, glycosylphosphatidylinositol anchor molecules are abundant in trypomastigote membranes and can induce the synthesis of cytokines such as tumor necrosis factor and interleukin–12 by stimulating TLR–2 [38].

**Fig 6:**
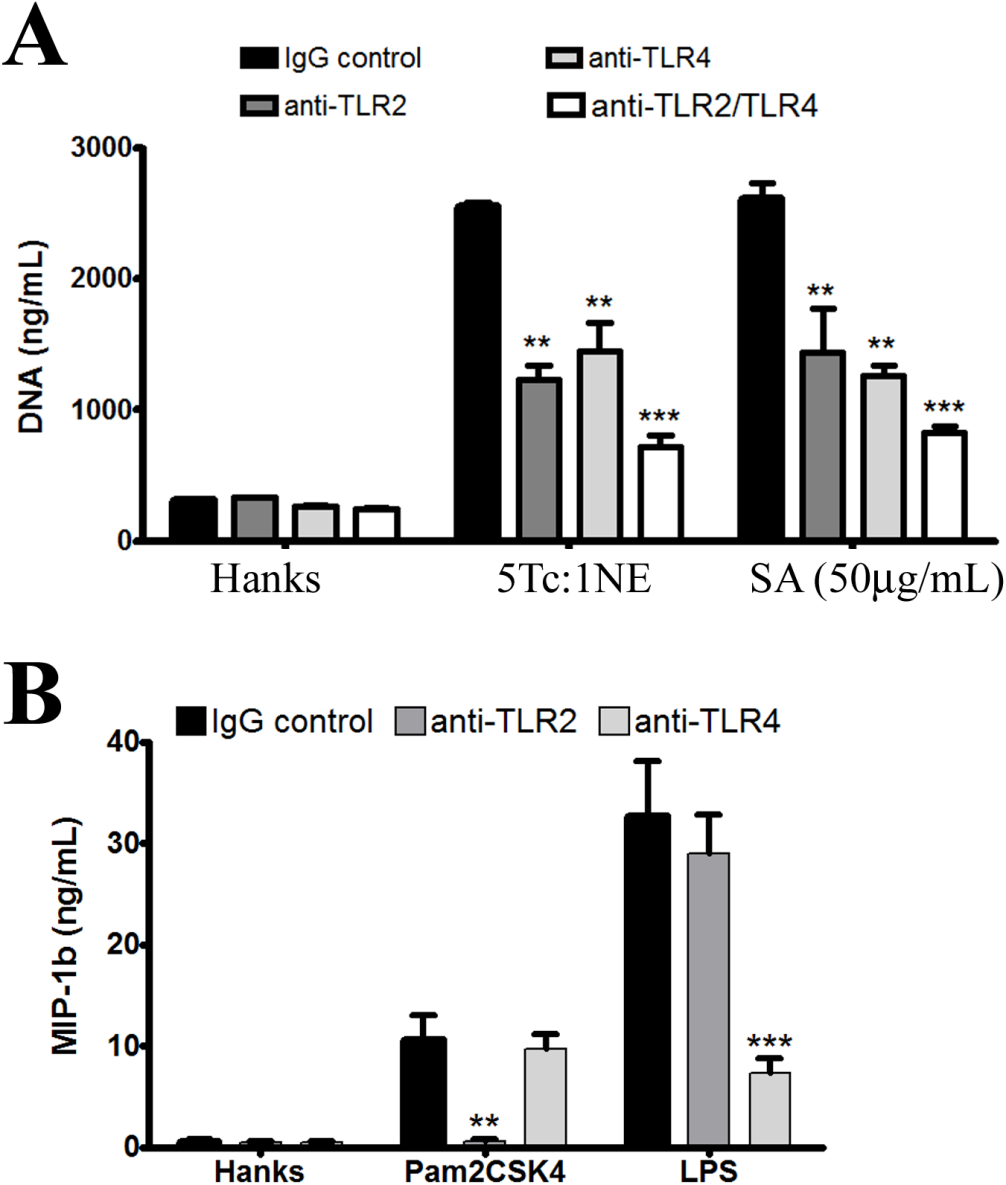
*Trypanosoma cruzi* parasites and their soluble antigens induce NET release by activating TLR–2 and TLR–4. **(A)** Neutrophils (2 × 10^5^) preincubated with monoclonal antibodies (anti-TLR2 and/or anti-TLR4) were co-cultivated with *T*. *cruzi* or its soluble antigens for 4 h and analyzed for NET release by extracellular DNA quantification. Neutrophils incubated with non-specific anti-human IgG were used as the negative control. DNA in the supernatant was quantified using the dsDNA High Sensibility Assay Kit. All experiments were conducted in triplicate in independent assays. (B) MIP–1β was measured in the supernatant from Pam2CSK4- and LPS-stimulated neutrophils previously blocked with monoclonal antibodies (anti-TLR2 and/or anti-TLR4). Neutrophils incubated with non-specific anti-human IgG were used as the negative control. The results were analyzed by ANOVA followed by Bonferroni multiple comparisons test. Asterisks indicates significant differences compared with the control group (IgG control for each treatment) (**P < 0.01).

Studies have demonstrated that the absence of TLR4 and TLR4-MyD88 signaling are associated with decreased release of DNA from neutrophils stimulated by *Haemophilus influenzae* [39, 40]. Other pathways involving TLRs are also important in the generation of NETs, such as TLR2 [40, 41]. In order to investigate whether TLRs are involved in NET release induced by *T*. *cruzi*, human neutrophils were blocked with specific antibodies against TLR2 and TLR4 molecules before incubation with *T*. *cruzi* parasites and their soluble extracts (**Fig 6**). The results showed that blocking of TLR–2 or TLR–4 significantly influenced the release of DNA promoted by both the parasite and its soluble extract. Blocking of TLR2 and TLR4 accentuated the decrease in NET release, but did not inhibit it completely. This result indicates that other receptors are involved in neutrophil stimulation by the *T*. *cruzi* parasite during NET release. The control IgG antibody did not alter NET release induced by *T*. *cruzi* or its soluble extract. Blocking by monoclonal antibodies was confirmed, as the presence of chemokine MIP-β was decreased in the supernatant of neutrophils stimulated with TLR-2- and TLR-4-specific agonists (Pam2CSK4 and LPS, respectively). Parasite molecules such as glycosylphosphatidylinositol anchors and their DNA may be responsible for stimulating the generation of NETs induced by *T*. *cruzi*, heat-killed *T*. *cruzi*, or its soluble antigens. The appointment of these pathogen-associated molecular patterns does not exclude the possibility that many other molecules present in the soluble extract of the parasite can participate individually or synergistically in the induction of NETs.

In conclusion, we demonstrated that neutrophils stimulated by *T*. *cruzi* generate NETs composed of DNA, histones, and elastase. The release occurs in a dose-, time-, and ROS-dependent manner, affecting the infectivity/pathogenicity of the parasite without affecting its viability. Living parasites are not mandatory for the release of NETs following stimulation of the TLR–2 and TLR–4 receptors, as the same results were obtained when the soluble extract from *T*. *cruzi* was tested. Thus, we suggest that *T*. *cruzi* parasite induces NET release from neutrophils by activating ROS production after interacting with TLR–2 and TLR–4.

Our data and those of others contribute to a better understanding of the stimulation of NETs by parasites. However, the relevance of these events during *in vivo* infection and whether parasites have also developed escape mechanisms to resolve NET, as are known for some bacterial species, should be further examined.

## Supporting Information

S1 Fig *Trypanosoma cruzi parasites and its soluble antigens* induce NET release containing histone and elastase. Neutrophils were incubated with *T*. *cruzi* (5 Tc: 1 Ne), soluble antigen (50 μg/mL), PMA (25 nM), or only HANKS for 4 h. NETs were observed by fluorescence staining using antibodies: anti-histone (green), anti-elastase (green), and fluorescein isothiocyanate-conjugated antibody and DAPI (blue). (40× objective)
(PNG)

S1 Movie Culture cell infected with *T*.*cruzi* trypomastigotes pretreated with HANKS. Blue arrows indicate infected cells. Trypomastigotes and amastigote forms can be observed in the supernatant.
(MP4)

S2 Movie Culture cell infected with *T*.*cruzi* trypomastigotes pretreated with NET solution. Blue arrows indicate infected cells. Trypomastigotes and amastigote forms can be observed in the supernatant.
(MP4)
