# Supplementary figures and images for: Trypanosoma cruzi and Its Soluble Antigens Induce NET Release by Stimulating Toll-Like Receptors
Source: PLoS One. 2015 Oct 2;10(10):e0139569. doi: 10.1371/journal.pone.0139569 (PMC4591979; doi:10.1371/journal.pone.0139569)

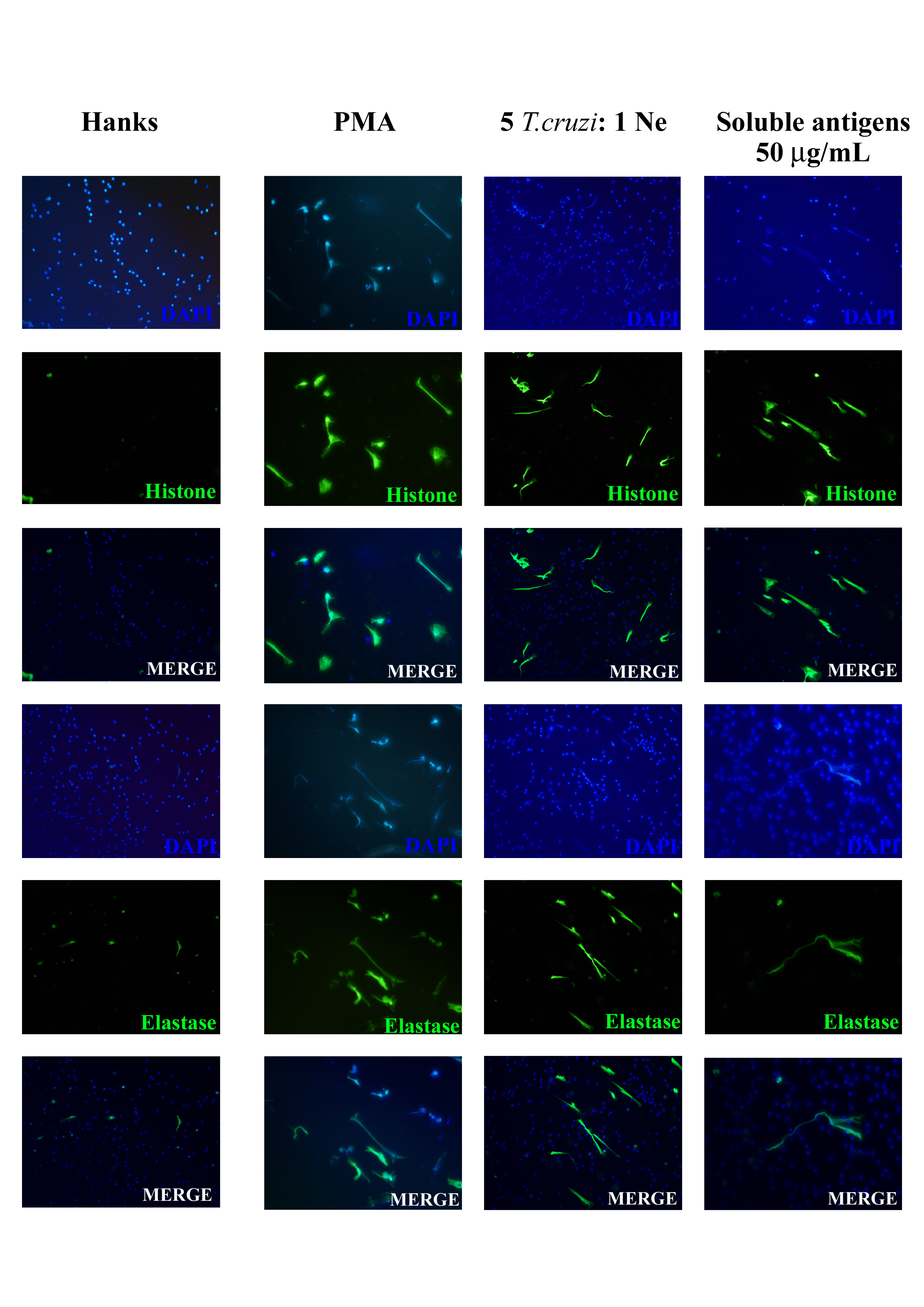

Supplement: S1 Fig — Neutrophils were incubated with T. cruzi (5 Tc: 1 Ne), soluble antigen (50 μg/mL), PMA (25 nM), or only HANKS for 4 h. NETs were observed by fluorescence staining using antibodies: anti-histone (green), anti-elastase (green), and fluorescein isothiocyanate-conjugated antibody and DAPI (blue). (40× objective) (PNG) [file pone.0139569.s001.png]
